# Supplementary figures and images for: Analysis of intestinal epithelial cell responses to Cryptosporidium highlights the temporal effects of IFN-γ on parasite restriction
Source: PLoS Pathog. 2024 May 8;20(5):e1011820. doi: 10.1371/journal.ppat.1011820 (PMC11078546; doi:10.1371/journal.ppat.1011820)

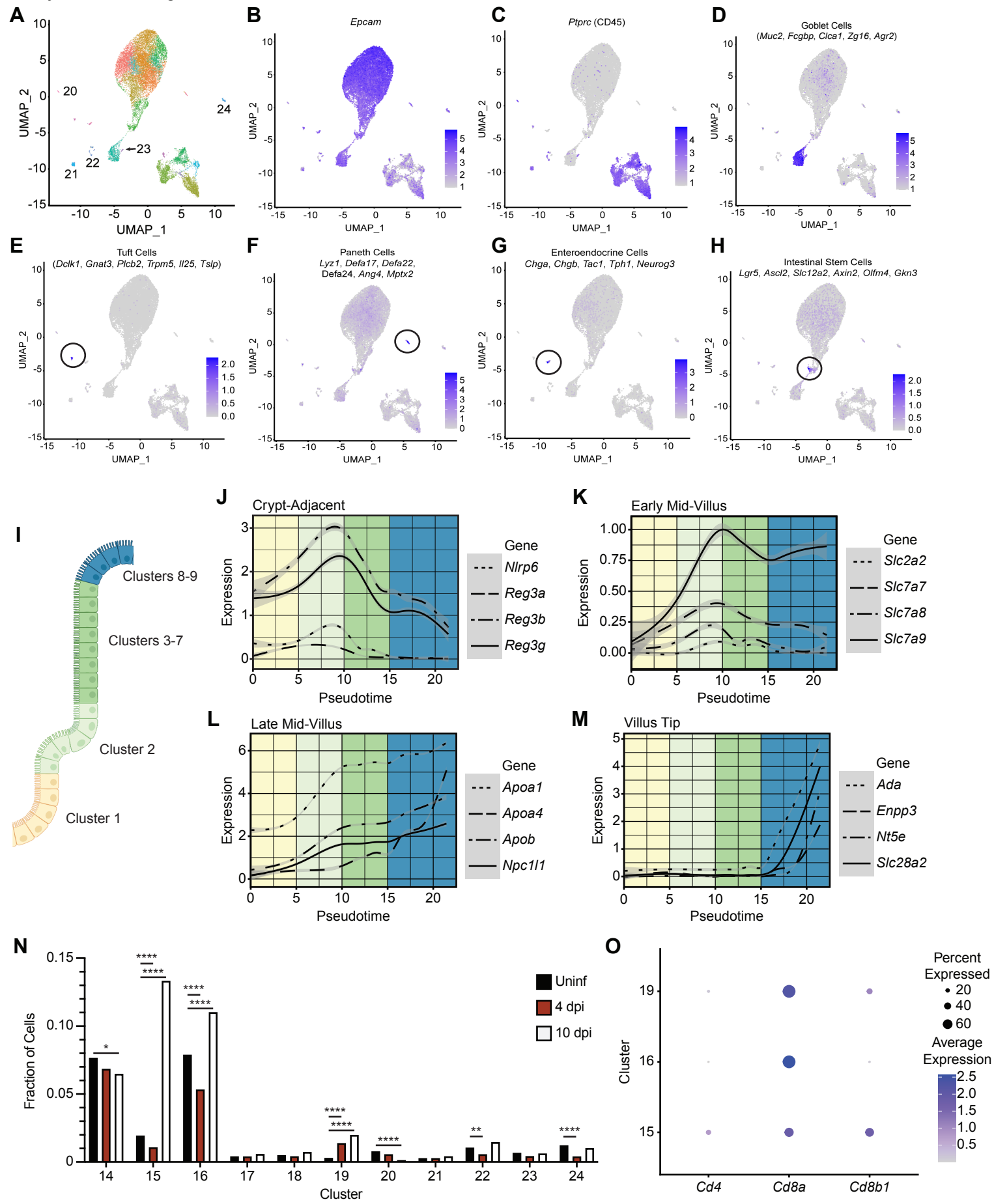

Supplement: S1 Fig — (A) Same UMAP as in Fig 1A with remaining clusters labelled. (B-H) Projection of gene expression or gene signatures on UMAP of IEC and IEL clusters to identify those that represent (B) Epcam+ IEC, (C) Ptprc+ (CD45+) IEL, (D) goblet cells, (E) tuft cells, (F) Paneth cells, (G) enteroendocrine cells and (H) intestinal stem cells. (I) Cartoon depicting where cells from each enterocyte cluster are projected to lie in the crypt and villus, coloured by pseudotime value. (J-M) Graphs depicting expression of marker genes for crypt-adjacent (J), early mid-villus (K), late mid-villus (L) and villus tip (M) enterocytes versus pseudotime. (N) Fraction of cells in clusters 14–24 separated by sample. (O) Dot plots of Cd4, Cd8a, and Cd8b1 expression in IEL clusters 15, 16, and 19. Data in (N) were analyzed by χ2 test between uninfected and 4 dpi and between uninfected and 10 dpi. *p < 0.05, **p < 0.01, ****p < 0.0001. (PDF) [file ppat.1011820.s001.pdf]

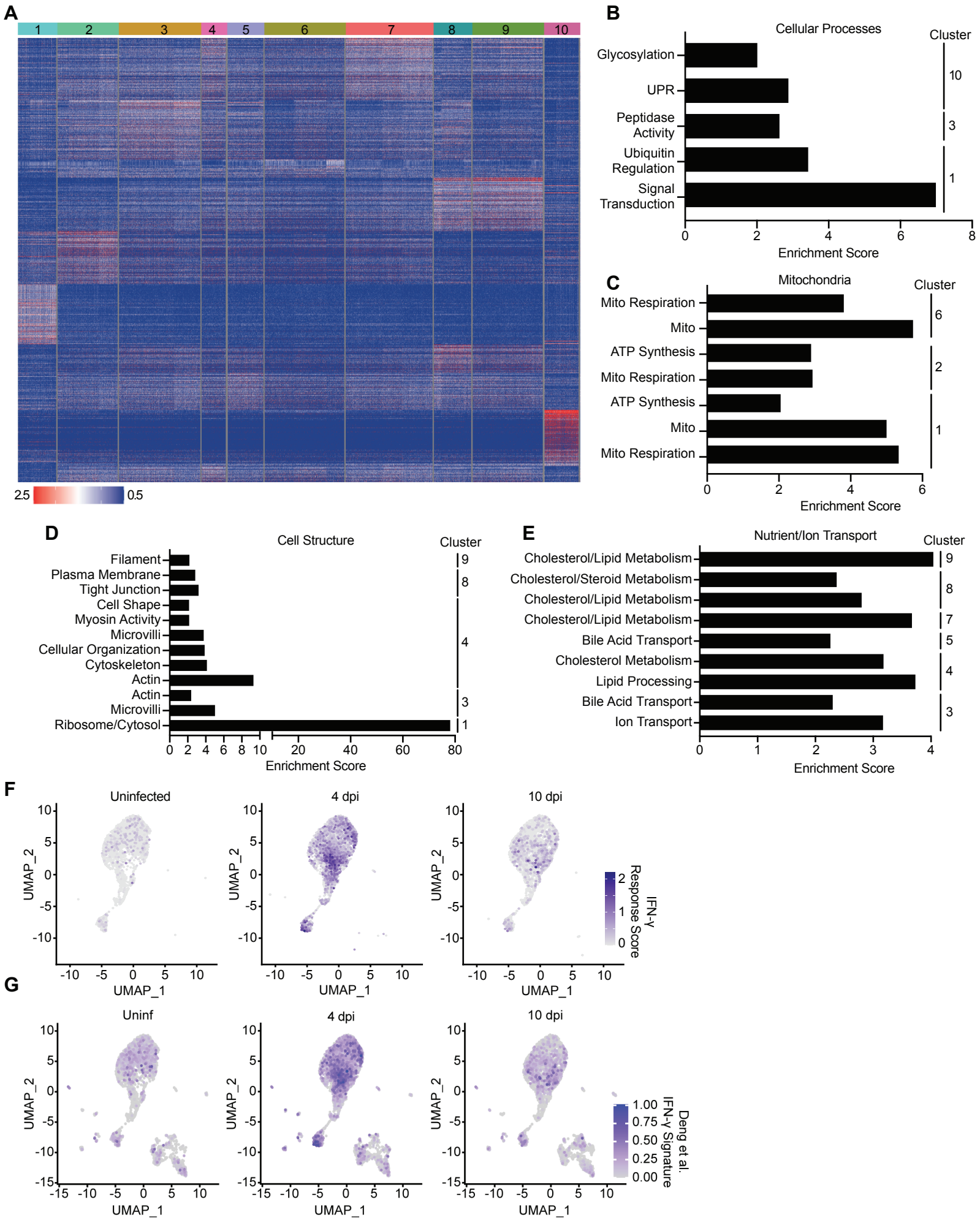

Supplement: S2 Fig — (A) Heatmap of top 100 marker genes for each IEC cluster. List available in S1 Table. (B-E) Database for Annotation, Visualization and Integrated Discovery (DAVID) was used to generate functional annotation clustering of the marker genes for each cluster. Functional annotations for the (B) “Cellular Processes”, (C) “Mitochondria”, (D) “Cell Structure”, and (E) “Nutrient/Ion Transport” categories are indicated. (F) IFN-γ signature score plotted on UMAP for enterocyte clusters, separated by time point. (G) Deng et al. IFN-γ signature score (generated using expression of Bst2, Stat1, Igtp, Irf8, Ifit1, Ifit3, Tbk1, Parp14, Gbp7, Irf1, Trim6, Dcst1, Cd40, Rab43, Mrc1, Cited1, Ifngr2, and Ido1) plotted on the UMAP for each sample. (PDF) [file ppat.1011820.s002.pdf]

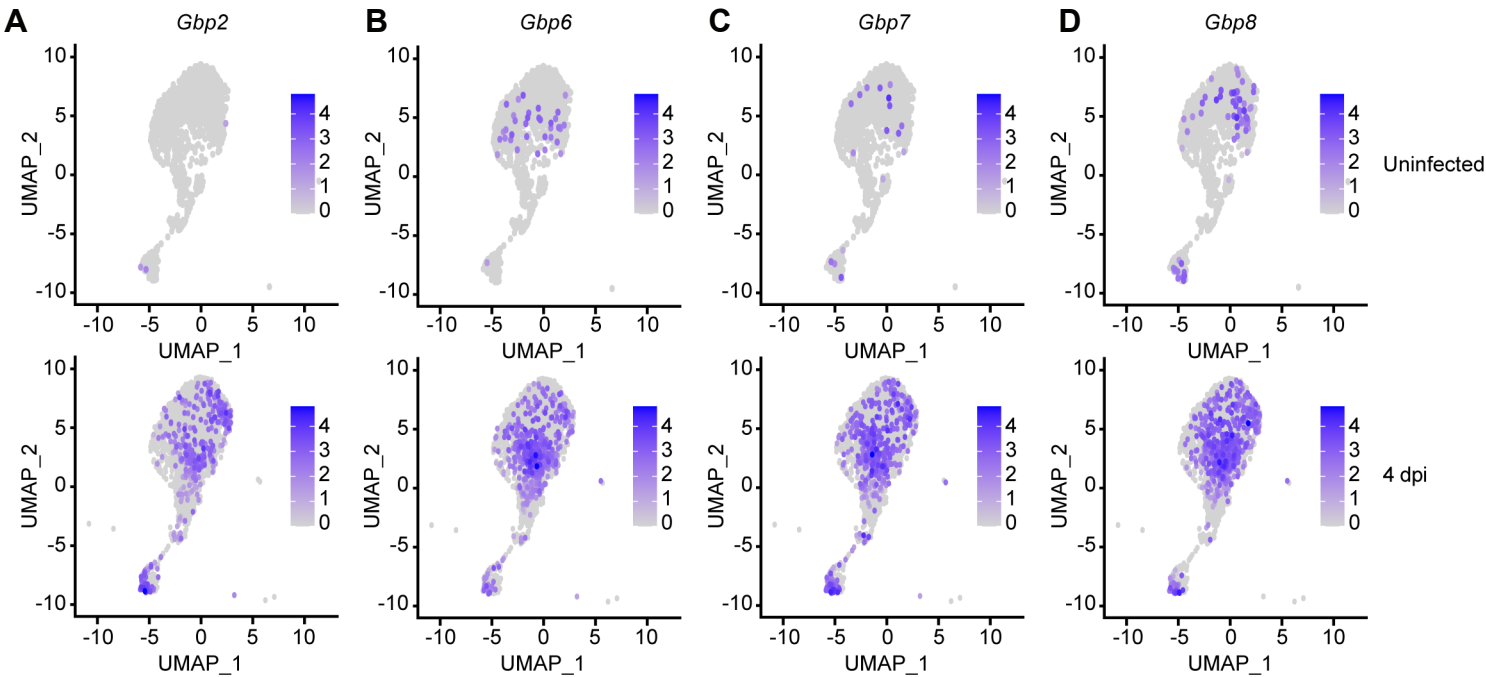

Supplement: S3 Fig — Expression of (A) Gbp2, (B) Gbp6, (C) Gbp7 and (D) Gbp8 projected onto UMAP of IEC clusters from the uninfected and 4 dpi samples. (PDF) [file ppat.1011820.s003.pdf]

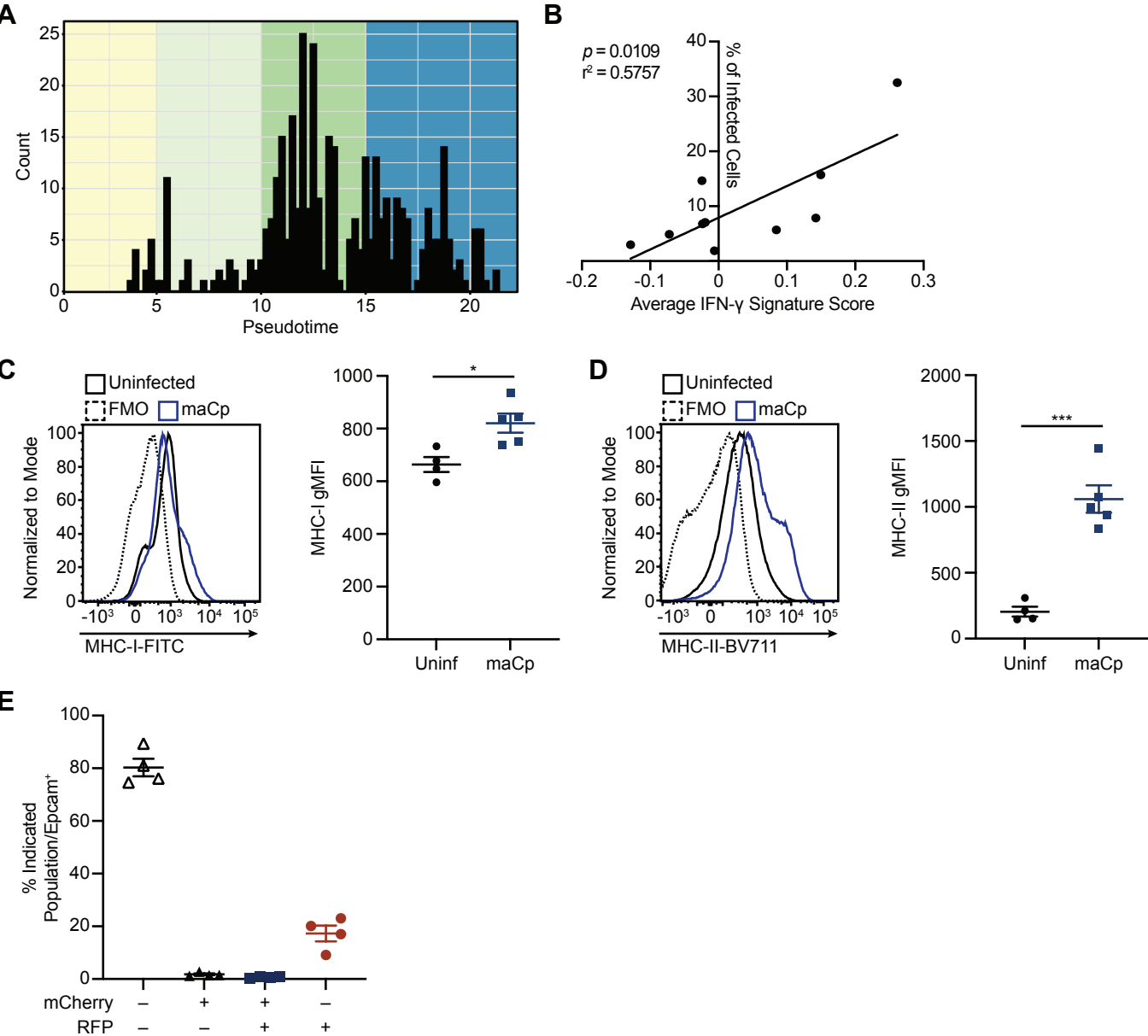

Supplement: S4 Fig — (A) Histogram of the distribution of infected cells from the 4 dpi sample across pseudotime. (B) Linear regression analysis of the percentage of infected cells in each enterocyte and goblet cell clusters versus the average IFN-γ signature score for cells in that cluster. (C-D) Representative histogram and gMFI of MHC-I (C) and MHC-II (D) in uninfected WT mice (black line), and WT mice infected with 5x10^4^ maCp oocysts (blue line). Dotted line on histograms indicates FMO control. (E) Frequency of mCherry-RFP-, mCherry+RFP-, mCherry+RFP+ and mCherry-RFP+ IEC from infected M1Red mice 5 dpi with 5x10e4 maCp oocysts. Data in (C-E) are representative of two independent experiments with n = 4–5 mice per group per experiment. Data in (C-D) were analyzed by two-tailed, unpaired Student’s t-test. Error bars indicate mean ± SEM. *p < 0.05, ***p < 0.001. (PDF) [file ppat.1011820.s004.pdf]

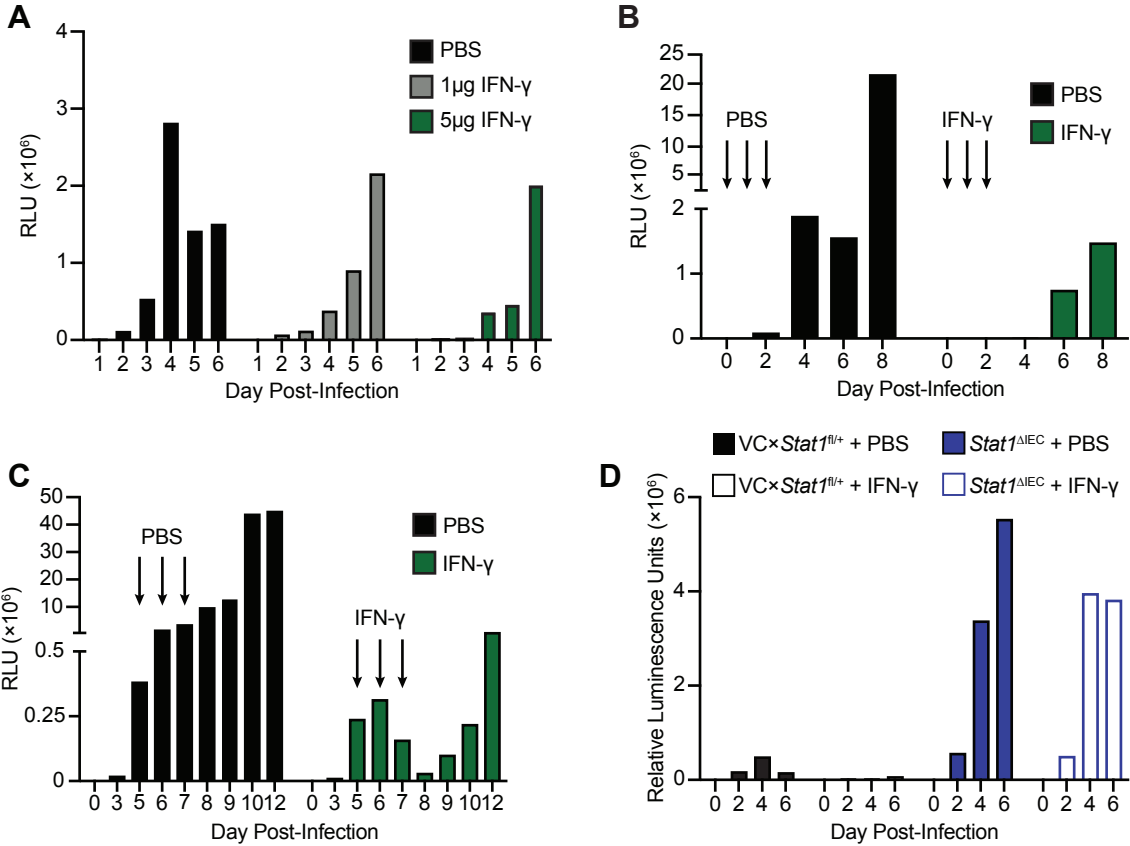

Supplement: S5 Fig — (A) Ifng-/- mice were treated with 1μg or 5μg of IFN-γ 6 hours prior to being infected with 104 maCp oocysts and nanoluciferase activity was used to measure fecal oocyst shedding. (B-C) Ifng-/- mice were infected with 104 maCp oocysts and nanoluciferase activity was used to measure fecal oocyst shedding. Mice were treated with PBS or IFN-γ 0, 1 and 2 dpi (B) or 5, 6, and 7 dpi (C). (D) Stat1ΔIEC mice and VCxStat1fl/+ heterozygote control mice were infected with 5x10^4^ maCp oocysts. One group of each genotype was treated with PBS or IFN-γ 0, 1 and 2 dpi and nanoluciferase activity was used to measure fecal oocyst shedding. Data in (A-D) are representative of two independent experiments, n = 3–5 mice per group, per experiment. (PDF) [file ppat.1011820.s005.pdf]

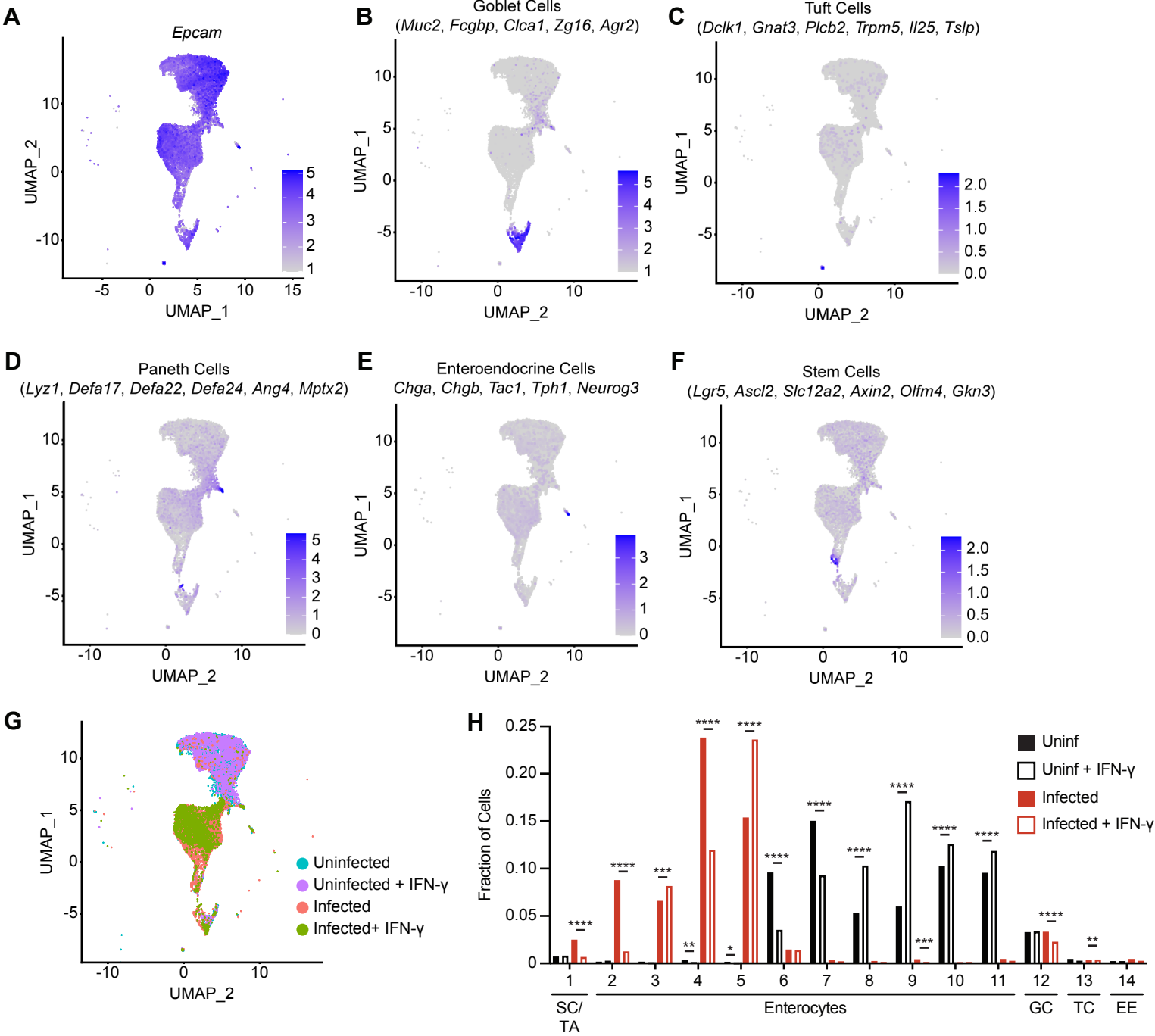

Supplement: S6 Fig — Projection of gene expression or gene signatures on UMAP clustering to identify clusters representing (A) Epcam+ IEC, (B) goblet cells, (C) tuft cells, (D) Paneth cells, (E) enteroendocrine cells and (F) intestinal stem cells. (G) UMAP of IEC clusters colored by sample identity (blue, uninfected; purple, uninfected + IFN-γ; orange, infected; green, infected + IFN-γ). (H) Fraction of cells in IEC clusters, separated by sample. Data in (F) were analyzed by χ2 test between uninfected and uninfected + IFN-γ, and between infected and infected + IFN-γ. *p < 0.05, **p < 0.01, ***p < 0.001, ****p < 0.0001. (PDF) [file ppat.1011820.s006.pdf]

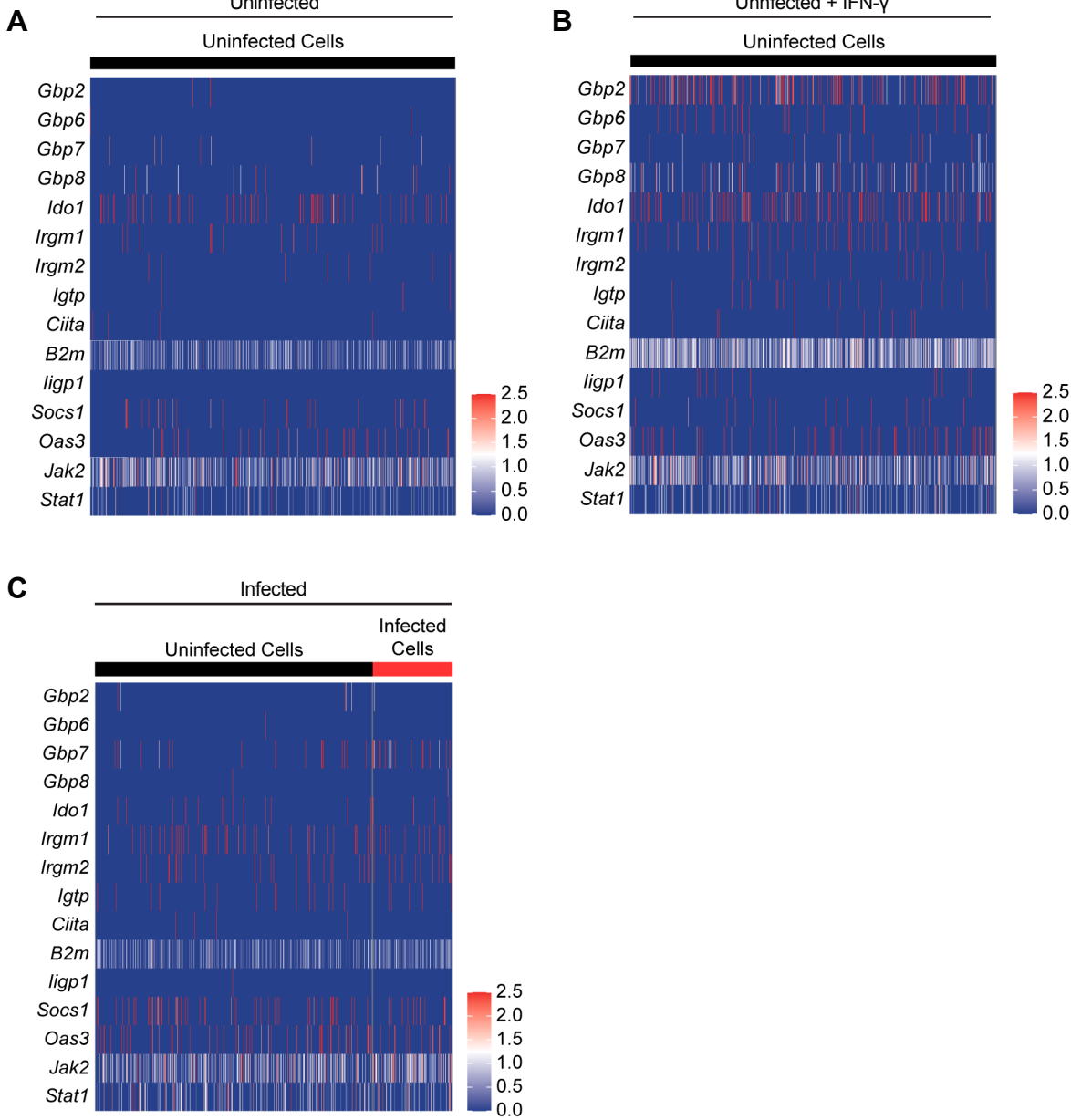

Supplement: S7 Fig — Heatmap of IFN-γ-stimulated gene expression in uninfected IEC from the (A) uninfected and (B) uninfected + IFN-γ samples. (C) Heatmap of IFN-γ-stimulated gene expression compared between uninfected and infected IEC from the infected sample. (PDF) [file ppat.1011820.s007.pdf]

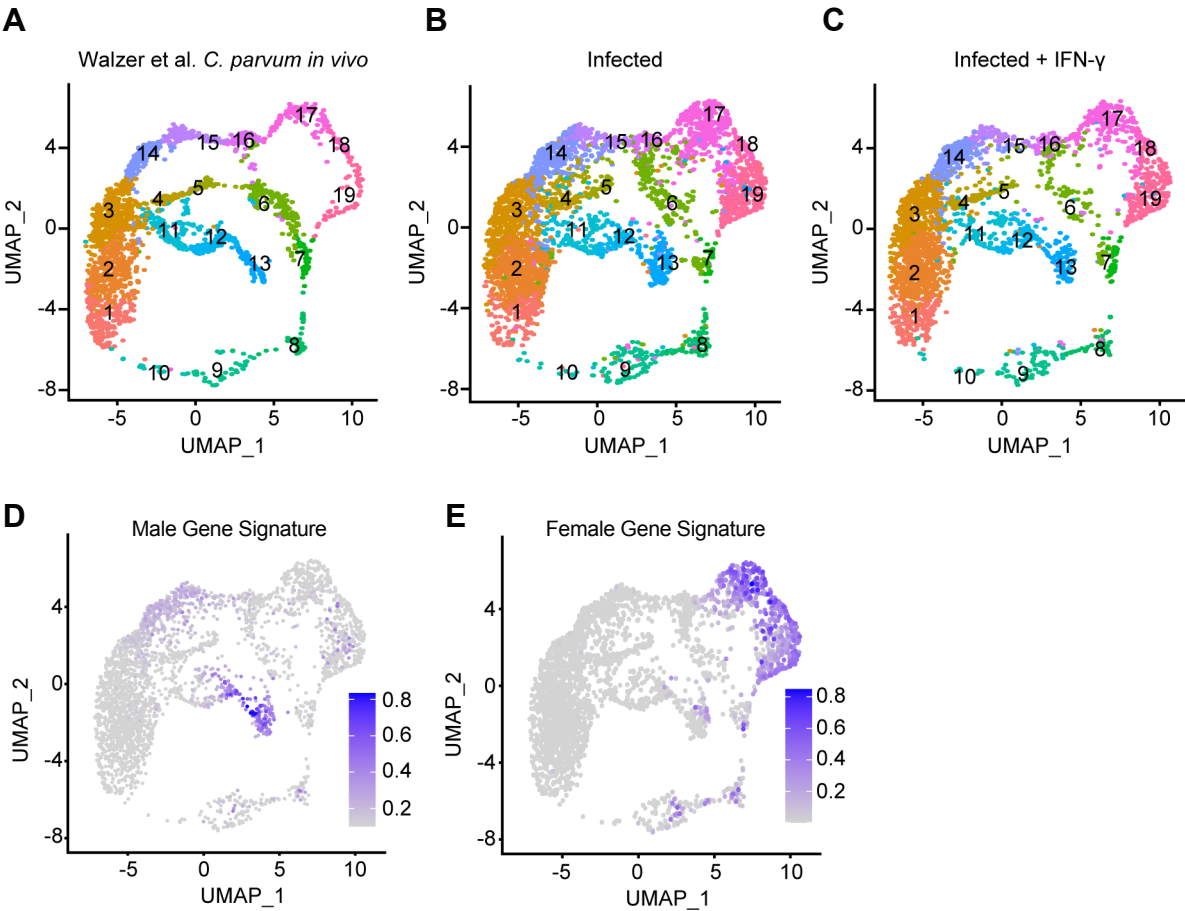

Supplement: S8 Fig — (A) UMAP clusters of scRNA-seq data from Ifng-/- mice infected with C. parvum (from Walzer et al.) [33] or maCp-infected mice treated with PBS (B) or IFN-γ (C) following alignment to the Cryptosporidium genome. (D) Male and (E) Female gene signature expression scores projected on UMAP clustering of scRNA-seq data from infected and infected + IFN-γ samples following alignment to Cryptosporidium genome. Genes used for each gene signature are in S3 Table. (PDF) [file ppat.1011820.s008.pdf]

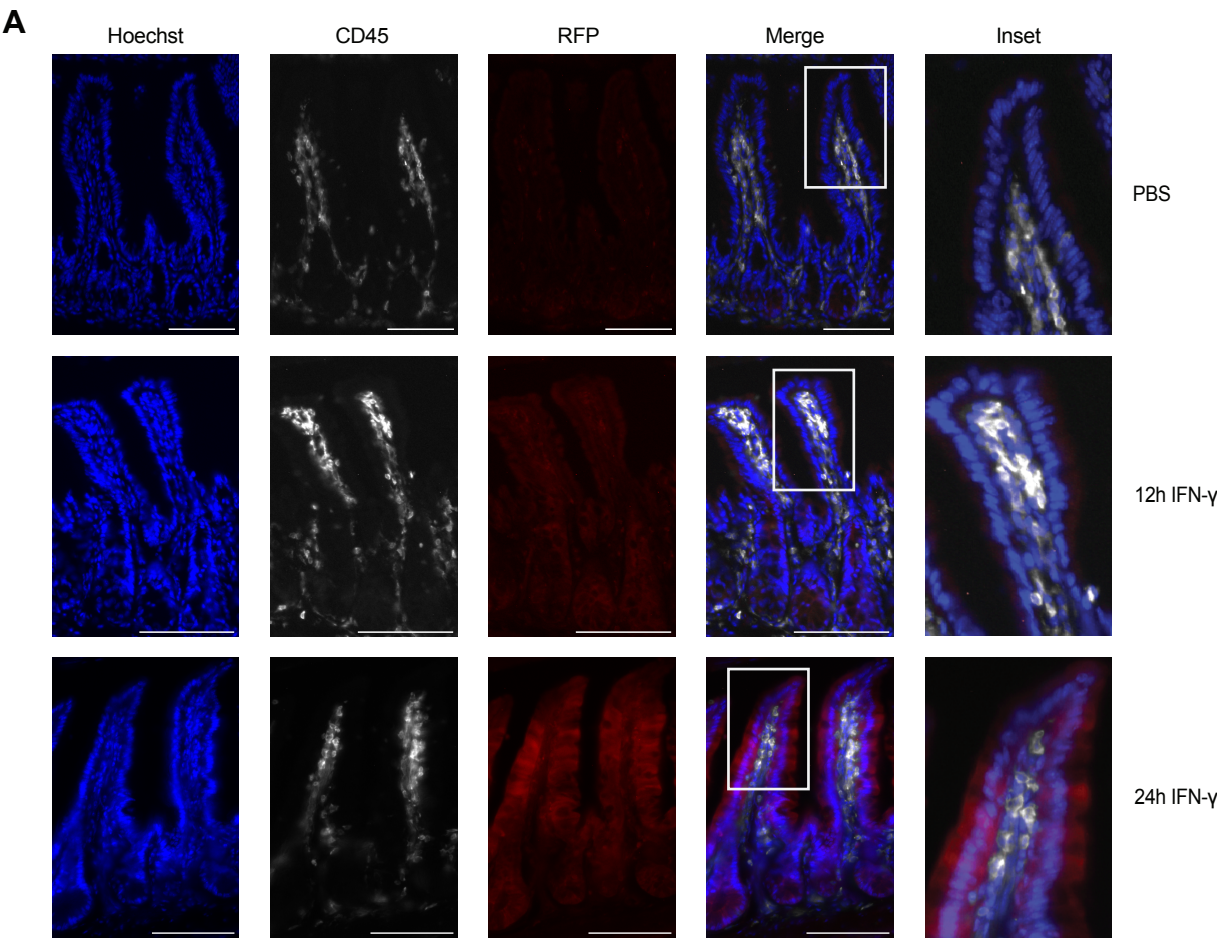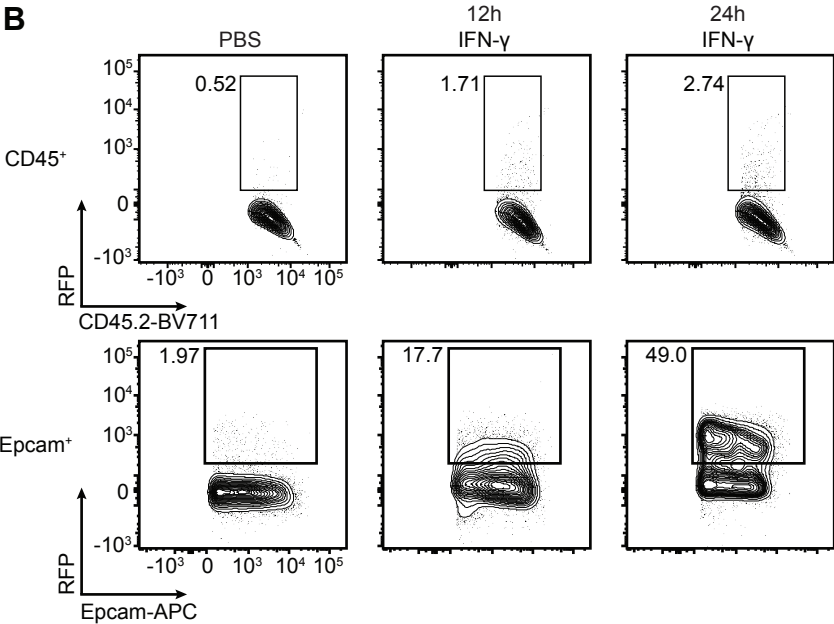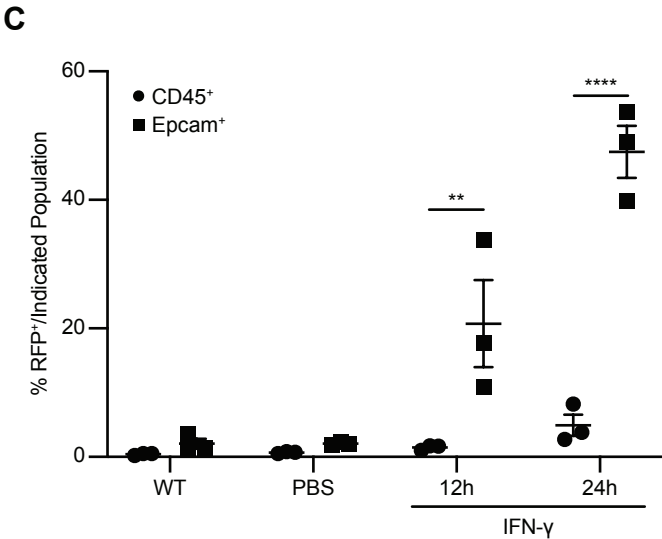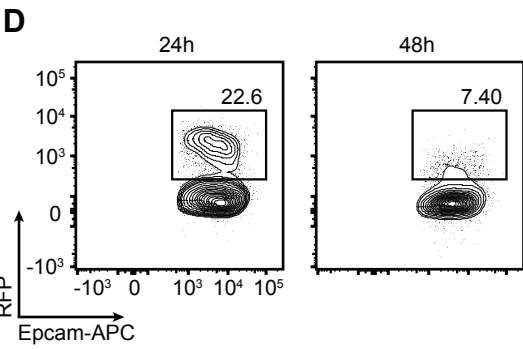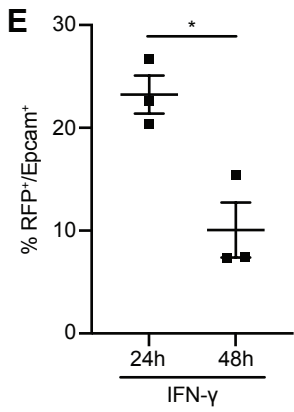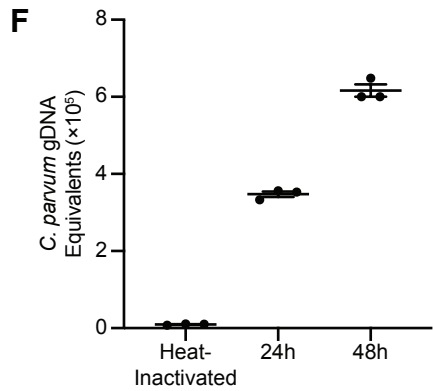

Supplement: S9 Fig — (A) Fluorescence microscopy images of RFP (red), CD45 (grey) and Hoechst DNA stain (blue) in ileal sections from M1Red mice treated with PBS or 12h and 24h post-IFN-γ treatment. Scale bar 100 μm. White box in merge is depicted under “inset”. (B) Representative flow cytometry plots and (C) frequency of RFP+ Epcam+ IEC and CD45+ IEL from M1Red reporter mice treated with PBS or IFN-γ 12h and 24h prior to analysis. (D) Representative flow cytometry plots and (E) frequency of RFP+ IEC 24h and 48h post-injection with IFN-γ. (F) C. parvum burden was assessed by qPCR 24h and 48h post-infection in ALI monolayers or in ALI monolayers infected with heat-inactivated C. parvum sporozoites. Data are presented as genomic DNA (gDNA) equivalents after comparison to a standard curve of Ct value versus number of oocysts. Data in (A-E) are representative of two independent experiments, with n = 3 mice per group, per experiment. Data in (F) are representative of two independent experiments with n = 3 replicates per group, per experiment. Data in (C) were analyzed by two-way ANOVA with Šidák’s post-test for multiple comparisons. Data in (E) were analyzed by two-tailed, unpaired Student’s t-test. Error bars indicate mean ± SEM. *p < 0.05, **p < 0.01, ****p < 0.0001. (PDF) [file ppat.1011820.s009.pdf]
