# Supplementary material for: Analysis of intestinal epithelial cell responses to Cryptosporidium highlights the temporal effects of IFN-γ on parasite restriction
Source: PLoS Pathog. 2024 May 8;20(5):e1011820. doi: 10.1371/journal.ppat.1011820 (PMC11078546; doi:10.1371/journal.ppat.1011820)
Supplement: Attachment — Submitted filename: Response to Reviewers_R1.docx [file ppat.1011820.s014.docx]

**Part I - Summary**

Reviewer #1: The manuscript by Pardy et al. investigated the role of the IFN-g signalling in intestinal epithelial cells during the infection of mice with Cryptosporidium parasites. The authors very nicely desribe the kinetics of IFN-g responsiveness in different IEC clusters during the Cryptosporidium infection.
Overall, the study is well-designed and generally the main conclusions of the paper are supported by the data presented. I hope my comments will be helpful to potentially improve the manuscript further.

Reviewer #2: The manuscript by Pardy et al using single cell transcriptomics and functional in vivo to decipher the role of IFNg signaling in mediating epithelial responses during Cryptosporidium infection. Overall, this is a well-done study that is well suited to Plos pathogens. However, I have a few concerns that should be addressed prior to publication

Reviewer #3: The study reported in the manuscript entitled “Analysis of intestinal epithelial cell responses to Cryptosporidium highlights the temporal effects of IFNg on parasite restriction” by Pardy et al., reports the effects of IFNg on intestinal epithelial cells in response to infection by Cryptosporidium.
The study by Pardy et al. illuminates the important role of IFNγ-mediated response in intestinal epithelial cells to Cryptosporidium infection. The study utilizes single-cell RNA sequencing, aligning transcript reads to both the mouse and parasite genomes. This approach allows the analysis of infected and bystander epithelial cells. These observations suggest that IFN-γ activation led to a robust decrease in oocyst shedding in enterocytes and goblet cells. Overall, this study provides insight into the global impact of infection with Cryptosporidium on IEC and suggests a model in which IFN-γ-mediated bystander activation of uninfected enterocytes is important to limit the Cryptosporidium growth cycle within its host. However, some refinement of the text (see examples below) and further analysis need to be addressed before the manuscript is ready for publication.

**We thank the reviewers for their suggestions to improve the manuscript. We have addressed their concerns as thoroughly as possible and hope the revised version is suitable for publication in *PLOS Pathogens*. Responses to specific points raised are in bold below.**

**Part II – Major Issues: Key Experiments Required for Acceptance**

Reviewer #1: Major issue:

It would be of interest if the authors could suggest /show how IFN-g-mediated bystander activation of uninfected entrocytes can come about.

**Previous work in other infectious systems has shown that local cytokine production can condition bystander cells (Perona-Wright et al. 2010). We have added data to the heatmaps in Fig 2D and 3F that shows ubiquitous IEC expression of *Ifngr1*, which highlights they are responsive to IFN-γ signalling regardless of infection status. This is also supported by the responsiveness of IEC to IFN-γ in uninfected M1Red mice (S9 Fig), when uninfected *Ifng*^-/-^ mice were treated with IFN-γ in the scRNA-seq data (S7A-B Fig), and the pre-incubation of intestinal organoid cultures with IFN-γ prior to infection (Fig 5E). We have also modified the introduction and the discussion to discuss why/how IFN-γ would be acting on uninfected IEC: “A recent study demonstrated that IL-18 release by infected IEC and IL-12 production by dendritic cells stimulates gut-resident ILC1 to produce IFN-γ (18). These data suggest a model in which areas of *Cryptosporidium* replication would be associated with local production of IFN-γ, which would be available to activate adjacent uninfected IEC. Indeed, in this study the strength of the IFN-γ signature in each IEC cluster correlated with the proportion of infected cells in that cluster.”**

In the Figures 3E and 3F, it was shown that infected and uninfected IECs showed comparable enrichment of the IFN-g response gene set. Could it be possible that infected cells transmit signals for epithelial defense program via gap junction signaling (Ca2+ wave spreading etc.) to the uninfected cells during the Cryptosporidium infection?

**We appreciate the comment and our data sets do not rule out a role for gap junction signalling. For example, there is a literature that links Ca2+/calmodulin dependent kinase (CaMK) II to enhancement of IFN-γ signalling through interactions with STAT1. However, as discussed in our previous response, ubiquitous IEC expression of the IFN-γ receptor (and associated signaling machinery) is likely the most important way by which IEC encounter IFN-γ to upregulate this gene set.**

Reviewer #2: • Throughout the paper enrichment scores for IFNg signaling are used without statistics to claim differences between groups.

**Thank you for this suggestion and indicators of statistically significant differences have been added to the figures and details on the tests run included in the figure legends. For the DAVID analysis, we have clarified in the methods that the functional annotations we report are only those with an enrichment score greater than 2, which corresponds to *p* < 0.01 (the enrichment score is calculated as the geometric mean of -log(*p*) for each member of the annotation cluster. Please see S2 Table for more details. We hope these additional details are sufficient.**

• The claim of bystander activation both in the abstract and text based on scRNAseq data and enrichment scores seems exaggerated. IF imaging of STAT1 in RFP parasite infected mice should help corroborate this claim or it should be textually toned down.

**We have modified the text and abstract by removing the word “bystander” and refer to the IEC simply as “uninfected IEC”. We believe the conclusion that IFN-γ activates uninfected IEC is demonstrated by the sequencing datasets. In particular, the observation that a single IFN-γ treatment activates ISG expression in uninfected IEC from maCp-infected *Ifng*^-/-^ mice (Fig 4D). This conclusion is also supported by experiments in uninfected mice, which show that IFN-γ activates ISG expression in IEC by scRNA-seq (S7 Fig) and fluorescent imaging in M1Red mice (S9 Fig). In particular, pre-incubating intestinal organoids with IFN-γ prior to infection is sufficient to activate anti-parasitic defenses in the IEC (Fig 5E).**

• The IF images are extremely hard to interpret given the poor resolution and lack of negative (uninfected and isotype) controls.

**We apologize for any difficulty interpreting the IF images. The use of fluorescently-tagged parasites to detect infected cells is a highly specific approach that has been well validated in culture and *in vivo*. We have included a higher power version of the infected goblet cell to Fig 3E to demonstrate that the fluorescent parasite is present within the cell and this also shows adjacent uninfected cells and goblet cells.**

• The text explaining figure 5 seems overly convoluted and could be simplified.

**We have modified the text for clarity.**

Reviewer #3: Comments:

1. A comprehensive enterocyte zonation analysis was conducted by Moor et al., 2018, showing the distribution of enterocyte function along the villus-crypt axis. While enterocytes at villus bottoms express an anti-bacterial gene program, the mid-villus enterocytes express absorption-related genes, and the villus-tip enterocytes express an anti-inflammatory program (the Cd73 immune-modulatory). How do these previous findings align with the pseudotime analysis performed in your study (shift in tasks upon infection)? And does the pseudotime reflect the enterocyte zonation correctly after the infection?

**This is a terrific suggestion and the study by Moor et al. is relevant to help analyze our data sets. Additional analyses have now been included that compare expression of genes associated with enterocytes that are crypt-adjacent, early mid-villus, late mid-villus, and at the villus tip. This further validates that the pseudotime analysis accurately reflects enterocyte progression (S1J-S1M Fig) and provides an improved way to present these data sets.**

**Since the focus of this submission is on how IFN-γ mediates parasite restriction, we did not include additional analysis of the impact of infection on enterocyte proliferation and function. These questions form the basis of a submitted manuscript that provides a more in-depth analysis of the impact of *Cryptosporidium* on epithelial cell proliferation. We hope the reviewer will appreciate that these additional studies fall outside the scope of the current submission.**

2. Related to this question, does the Cryptosporidium infection correlate with the mid-villus enterocytes reported in that study? And why does Crypto not infect the top villus compartment?

**Based on our pseudotime analysis and new data that demonstrates the location of infected cells in pseudotime, we have added the following to the manuscript: “Analysis of the distribution of infected cells across pseudotime similarly showed that parasites were mainly found in mid-villus and villus tip enterocytes (S4A Fig).”**

3. The proportion analysis lacks statistics and should show each mouse on the bars of each cell type to assess reproducibility.

**We have added statistical analyses for the proportion graphs, and multiple other graphs from the scRNA-seq analysis, with specific tests listed in the figure legends. We would also note that sequencing was done on pooled cells from two mice per group.**

4. The conclusion of the single cell analysis in Figures 1 and 2 (lines 189-190) needs to be refined or further examined. To me, it makes sense that the enterocytes gain IFNg program and are not generated or expended from the stem cells, as the proportion analysis showed no effect on stem/progenitor cells.

**Thank you for this suggestion, we have clarified this conclusion as follows: “Together, these data sets emphasize that *Cryptosporidium* infection results in the emergence of an IEC population associated with IFN-γ signalling.”**

5. A known mechanism to prevent parasite infection is the “Weep and Sweep” phenomenon. A more comprehensive examination of the gut status is needed. Is the tissue more proliferative? And results in higher enterocyte differentiation? Using the single cell data, it is possible to examine and then validate it via tissue stains.

**The “weep and sweep” mechanism is perhaps most prominently linked to the clearance of helminth infections but its relevance to Cryptosporidium is unclear. It has previously been described that *Cryptosporidium* infection leads to increased mitoses in IEC and pathological changes such as crypt and IEC hyperplasia (Lumadue et al. 1998; Abdou et al. 2013; Sateriale et al. 2019). While the current manuscript focuses on the impact of IFN-γ on enterocytes, the impact of *Cryptosporidium* on enterocyte turnover and differentiation is the focus of a recently submitted manuscript, which includes experimentally modifying these processes. We hope the reviewer will appreciate that these additional studies fall outside the focus of the current submission.**

6. Related to the previous comment, do goblet cells secret more mucins to prevent the attachment of Crypto?

***Cryptosporidium* is not typically associated with goblet cell hyperplasia or enhanced mucus secretion, which is supported by our data sets (we do not observe expansion of goblet cell populations during infection). However, we believe the imaging and sequencing data in this manuscript (Fig 3C-D) show they are infected by the parasite.**

7. Parasites elicit type II immunity. Do you identify ILCs type II or Th2 cells under the infection? and any role of IL-13, IL-4, or IL-5 in the clearance of the crypto?

**Parasitic helminth infections are associated with Th2 responses, whereas parasitic protozoan infections are most closely linked to Th1 responses. For *Cryptosporidium*, the immune response is dominated by IFN-γ production by multiple cell types including type I ILC and the induction of Th1 cells (McDonald et al. 1992; Griffiths et al. 1998; Russler-Germain et al. 2021; Gullicksrud et al. 2022). To better describe the cytokines and T cell polarization induced during infection, we have modified the introduction as follows: “Initial studies using *C. parvum* identified the importance of T cells and the cytokines IFN-γ, IL-12, and IL-18 for infection control (3, 12-15). Subsequently, it has been shown that IL-12 has a crucial role in the polarization of T helper 1 cells (16, 17), while local IL-12 and IL-18 drive type 1 innate lymphoid cell (ILC1) and natural killer cells to produce IFN-γ (18).”**

**Regarding IL-13 and IL-5, a specific role has not been described, although accumulation of low numbers of Th2 cells have been observed following *Cryptosporidium* infection and mice deficient for IL-4 exhibit delayed parasite clearance, but the basis of this observation is unclear (Aguirre et al. 1998; Russler-Germain et al. 2021; Pardy and Wallbank et al. 2024).**

8. Please discuss the difference of Crypto in shifting T cells’ response to Type I and not Type II immunity.

**We have modified the introduction to better describe the induction of type 1 immunity following *Cryptosporidium* infection, which is described above.**

9. What type of immune cells are elevated in IEL compartment?

**We have added analysis of marker gene expression (S1 Table) and specific T cell markers (S1O Fig) to the manuscript to show that the IEL clusters that are increased 10 dpi include canonical CD4^+^ T cells (cluster 15) and CD8αβ^+^ T cells (clusters 15 and 19), as well as CD8αα^+^ IELs (cluster 16), and describe these changes in the text.**

**Part III – Minor Issues: Editorial and Data Presentation Modifications**
Please use this section for editorial suggestions as well as relatively minor modifications of existing data that would enhance clarity.

Reviewer #1: Minor issues:


When adjusting the manuscript, the authors may want to keep the following aspects in mind:

On the page 7, the authors mention that the number of T cells recruited to the epithelium was increased at day 10 post-infection. Did they analyzed the composition and potential heterogeneity of those T lymphocytes. Are there more CD4+ or CD8+ T cells recruited to the epithelial cells? Do they exhibit cytotoxic phenotype?

**The major focus of this manuscript was the impact on epithelial cells, and so we limited discussion of changes in the T cell population. However, we have added broad analyses of these clusters to the text to show they are mostly non-canonical T cell populations (e.g. CD8αα+ IELs) and CD8αβ+ T cells, with a smaller population of CD4+ T cells (S1 Table and S1N Fig). Granzyme A and B are among the marker genes for each of the clusters that are increased at 10 dpi (clusters 15, 16, and 19; S2 Table).**

Several times, authors show the expansion of ISG enterocytes (clusters 2, 4 and 8) during Cryptosporidium infection, but there is also increase in IFN-g response score in goblet cells (cluster 10). Can authors speculate how important goblet cells (that are also associated with upregulated ISGs) for the clearance of the infection are? What about tuft cells?

**A potential role for goblet cells in control of *Cryptosporidium* has not been described. While imaging and sequencing data in this manuscript show they are infected by the parasite, there are no major changes to goblet cell populations. We have added the following to the discussion: “Goblet cells, which were infected by *Cryptosporidium* and upregulated ISG expression, do not have a well-described role in control *Cryptosporidium*, but in other experimental systems have been described to “pass” luminal antigen to local dendritic cells in the small intestine (44).” A role for tuft cells is an interesting question, although the data sets herein show they are not expanded during infection and we have confirmed this by IHC performed with Dr. Jakob von Moltke (University of Washington).**

On the page 10, authors show that the mice with defective IFN-g signaling in IECs displayed heightened levels of oocyst shedding, which was sustained for two weeks post-infection. In contrast to animals completely lacking IFN-g, mice lacking IFN-g signaling in IECs were able to clear infection. Does it mean that IFN-g signaling in macrophages and maybe in T cells is more essential to combat the Cryptosporidium infection? Could the authors discuss this possible “IFN-g-signalling hierarchy”?

**We have added the following on lines 232-235 to acknowledge the difference in phenotypes between mouse lines, and the implication that there may be indirect means by which IFN-γ signaling to dendritic cells or macrophages influences parasite clearance: “While a previous study demonstrated that *Stat1* in macrophages and dendritic cells is dispensable for parasite control (18), these data could suggest a role for IFN-γ signalling to immune cells in the clearance of *Cryptosporidium*.”**

Reviewer #2: • The figure presentations could be improved to ensure readability. For example, Figure 1 the scRNA seq clusters are labeled with numbers and only partially annotated. The genes used for annotation and not clearly noted and many clusters remain unannotated. Why not subcluster the epithelial cells and annotate?

**Thank you for this suggestion and we apologize that some of the data sets were unclear. We have labelled major IEC and IEL clusters in Fig 1A to ensure the figure was not overcrowded. We have added the same UMAP to S1 Fig to indicate the cluster numbers of the remaining clusters. Genes used for cluster annotation can be found in S1 Table (newly added), S2 Table and S1 Fig. We have also clarified in the text that epithelial cell sub-populations are annotated in the data set on lines 142-146: “Analysis of IEC clusters for cluster-specific marker gene expression (S2 Table) revealed that the majority of cells sequenced were enterocytes that separated into 8 sub-clusters (clusters 2-9), but also less common cell types such as intestinal stem cells/transit amplifying cells (cluster 1), goblet cells (cluster 10), tuft cells (cluster 11), enteroendocrine cells (cluster 12) and Paneth cells (cluster 13) (Fig 1A and S1D-S1H Fig).”**

• F images are also hard to interpret as

**We have added a higher power image of an infected goblet cell to improve clarity (Fig 3E) and have modified the text and figure legends to ensure the descriptions are as clear as possible.**

• The methods section is lacking any information about how epithelia were isolated and which fraction of epithelia (cypt/villi or both) were included in the analysis

**We have moved the isolation procedure and description of the cells isolated (mostly IEC/IEL, with a small population of crypt-derived stem cells) to its own section in the methods, with the sub-heading “IEC Isolation”, for improved clarity.**

• citations from the Yilmaz, Jabri and other lab have uncovered a role for MHCII expression in IECs should be included

**Thank you for bringing these references to our attention, we have cited them in the discussion as follows: “Previous studies have also shown that MHC-II expression is increased on IEC exposed to IFN-γ (41, 42), with pro- and anti-inflammatory roles ascribed to this pathway (41, 43-46)”**

Reviewer #3: Minor Comments:

1. Line 77, enterocytes express antimicrobial peptide as well.

**We have adapted the text to state: “The most common cells in the intestinal epithelium are enterocytes, tall columnar cells that mediate nutrient uptake and express antimicrobial peptides…”**

2. Line 147, the figure number is wrong. Also, presenting the pseudotime analysis of the three conditions (Control and infected, day 4 and 10) would really help to show the enterocyte differentiation state in each condition.

**We apologize for the confusion and have called out Fig 1B and Fig 1C as well as S1G Fig, which indicates where the stem cells are found. While changes to enterocyte turnover and barrier repair during infection is a question we are interested in, we have opted to omit that analysis as it forms the basis for another submitted manuscript.**

3. Line 158, as with the epithelial cells, the change in cell proportions is need to be statistically evaluated. Which T cells are elevated and what about ILCs? I wonder if most T cells are Th1 and not Th2 cells.

**We have added statistical analyses of changes in proportions to the graphs as suggested. Although we have focused our analysis on the IEC populations in the manuscript, there are few identifiable ILCs in the data set. We have added analyses of T cell clusters that are increased 10 dpi to show they correspond with CD8αα+ IELs, CD8αα+ T cells and CD4+ T cells. A more in-depth analysis of the T cell responses to Cryptosporidium can be found in Haskins et al. 2024. *Mucosal Immunol*. and Cohn et al. 2023. *Biorxiv*.**

4. The heatmaps in the study are hard to assess, so it may be beneficial to explore alternative data visualization tools, such as dot plots, to present specific genes.

**While we appreciate the suggestion, we chose to use heatmaps for larger lists of genes to better show a signature across clusters or infection status, which also captures variability in the data set.**

5. Fig. 2D and E-G, maybe dot plots will serve you better, and a violin plot to show the signature?

**We considered this option, however, not every IFN-γ-stimulated gene is expressed in every cell in the ISG enterocyte clusters, and certain plot types like violin plots could inadvertently under-represent the signature. As such we have chosen to use UMAP projection to help show that these genes are part of a signature that is concentrated in the ISG enterocyte clusters.**

6. As for the additional single cell paper (Ref. 29), what were the infection percentages (Figure 4B vs Figure 3A)? It seems higher than the one reported here and spans more enterocyte clusters. Why is that? Do these enterocyte states after infection correlate with this study?

**We apologize for the confusion – the data presented in Figures 4B and 3A are from two separate scRNA-seq experiments that were conducted for the present manuscript. The sequencing data in Reference 33, which defines the transcriptional atlas of *Cryptosporidium*, was exclusively used to understand the life stage of the parasites from infected cells shown in Fig. 4B. Nonetheless, as suggested the percentage of infected cells from IFN-γ KO mice (Fig 4B) is much higher than from the WT mice in Fig 3A, due to the crucial role of IFN-γ in control of *Cryptosporidium* and the uncontrolled parasite replication that occurs in its absence.**

7. As mentioned in the main comments, do the authors notice a change in epithelial proliferation or deepening of crypts, as stated in the discussion? These are important epithelial features associated with crypto and other gut infections that are not discussed in the results section. How does the IFNg treatment affect these features and the delayed enterocyte IFNg responses?

**The enterocyte extraction protocol used for this study predominantly yields mature enterocytes, with a small population of crypt cells, which limits analysis of these populations. Nonetheless, we do not observe major changes to crypt cells during infection in these data sets. The short term IFN-γ treatment did not cause overt changes to enterocyte populations based on the UMAP in Fig 4B, however we plan to pursue additional studies on this topic.**
